# Investigation of Scanning Strategies and Laser Remelting Effects on Top Surface Deformation of Additively Manufactured IN 625

**DOI:** 10.3390/ma15093198

**Published:** 2022-04-28

**Authors:** Alexandru Paraschiv, Gheorghe Matache, Nicolae Constantin, Mihai Vladut

**Affiliations:** 1 Special Components for Gas Turbines Department, Romanian Research and Development Institute for Gas Turbine COMOTI, 220D Iuliu Maniu, 061126 Bucharest, Romania; gheorghe.matache@comoti.ro (G.M.); mihai.vladut@comoti.ro (M.V.); 2 Materials Science and Engineering Faculty, University “Politehnica” of Bucharest, 313 Splaiul Independentei, 060042 Bucharest, Romania; nicolae.constantin@upb.ro

**Keywords:** LPBF, edge effect, corner effect, hatch angle, contour, remelting

## Abstract

The main drawbacks of the Laser Powder Bed Fusion (LPBF) process are the surface quality and dimensional accuracy of manufactured parts due to the edge and corner effects. These effects can be diminished by using an appropriate balance of the process parameters and scanning strategies. This paper focuses on the assessment of reducing the edge and corner effects that occur in additively manufactured IN 625 alloy via the LPBF technique by varying the hatch angle rotation (by 45°, 67°, and 90°) and volumetric energy density (VED), and using the laser top surface remelting technique (LSR). The edge and corner effects of the cubic samples were quantitatively evaluated on the top surface by 3D laser surface scanning. It was found that the edge and corner effects became more pronounced in the cases of samples built with no contour and hatch angles of 45° and 67°, while the smallest deformations were obtained when the hatch angle was rotated by 90°. Moreover, the heights of both the edge and corner ridges increase as the number of remeltings passing the top layer increases. Conversely, when a lower VED was used for melting the top layer(s) of the samples, the edge and corner ridges were slightly reduced.

## 1. Introduction

The Laser Powder Bed Fusion (LPBF) has gained considerable attention in the last decade due to its many advantages compared to conventional subtractive manufacturing techniques, such as geometric freedom, low product times, mechanical performances, and reduction of components and material flexibility [1,2,3,4,5]. For this reason, LPBF is one of the most promising metallic additive manufacturing (AM) techniques for producing end-use parts in the aerospace, biomedical, and automotive industries [2,4,6,7]. Nevertheless, despite progress in mechanical performance and material flexibility, there are still multiple drawbacks of LPBF technology—including anisotropy of the mechanical properties [8,9], high-temperature gradients [10], internal stresses [7], part distortion [7], low surface quality [1,5,6], and dimensional accuracy [11,12,13,14]—that keep it behind conventional manufacturing as a widely used technology. In an LBPF process, many physical phenomena occur, such as melting solidification, heat convection and radiation, Marangoni convection, absorption, recoil pressure, capillary forces, phase transformation, evaporation, and chemical reactions [9,15,16,17]. The surface quality and dimensional accuracy depend on many factors, such as material, powder particle size, laser parameters, layer thickness, building orientation, or chamber conditions [12,18,19]. Alongside the laser parameters and layer thickness, the scanning strategy strongly influences the thermal gradient, melt pool morphology, and thermomechanical behaviour of the additively manufactured (AMed) part [7]. Typical top and side surface defects encountered on the LPBF parts are high surface roughness, pores, thermal cracking, delamination, balling, and staircase effects [6,12,20]. Additionally, the elevated edges, often called the “edge effect”, affect the final dimensional accuracy and surface quality of LPBF parts [7,19], and can affect the manufacturing process and mechanical properties of the part due to contact between these edges and re-coater [5,12]. The edge effect is not only encountered in the LBPF process but also in other powder bed fusion processes such as laser engineered net shaping (LENS) and electron beam melting (EBM) [5]. The resultant thermal deformation issue of AMed parts during material solidification layer-by-layer is due to the high-temperature gradients and high cooling rates [10]. Expensive and time-consuming post-processing operations are used beforehand to improve the surface characteristics and make the LPBF parts suitable for many applications [6]. Therefore, a comprehensive analysis should be carried out at the initial step of designing the part to reduce the surface deformations and defects of AMed parts as much as possible. Depending on requirements, various surface finishing methods—such as mechanical methods (machining and abrasive blasting), chemical and electrochemical processes or thermal processes—are generally used for geometrical corrections and to reduce the surface roughness of AMed parts [12,18,21,22,23,24]. The influence of process parameters on the quality surface, especially surface roughness, has been investigated in many studies [2,3,6,11,24]. However, only a few studies have evaluated the edge effects and dimensional accuracy [5,19,20]. Many studies in recent years have shown that the surface quality can be improved by using the laser polishing of laser remelting technique (LSR) [1,11,12,20,23], appropriate scanning strategies [7,25], and laser powers [12,19].

Using the contour scan with an appropriate selection of parameters improves the inclined surfaces of parts but is not significant for up-skin surfaces [12]. LSR, whereby a melt layer is remelted one or several times, is considered by many [1,12,24,26,27] as a viable surface finishing method of LPBF parts. Using the LSR strategy for each layer of an AMed part, several characteristics and mechanical properties can be improved, including density [24], mechanical properties [28], surface roughness [29], friction and wear behaviour and microhardness [30], corrosion resistance [31], and microstructure [11,13,23]. By laser melting each layer of the LPBF parts, Yasa et al. [11] showed that the surface roughness and microhardness could be significantly improved, Liu et al. [1] revealed that the quality and relative density could be efficiently improved, and Shiomi et al. [32] found that residual stress of the as-built parts is reduced. However, improving the surface roughness and especially the density of parts is done by remelting each layer [1], which increases production times [29].

When laser remelting is used for the outer skin or the last layer of an additively manufactured part, the production time is not significantly affected, and surface characteristics such as surface roughness or balling effect can be reduced [13,20,23,24,27]. Ukar et al. [27] showed that the surface roughness of Selective Laser Sintering parts could be reduced by over 80%, and Kruth et al. [23] reduced the surface roughness (Ra) from 12 µm to 1.5 µm after using the laser remelting technique. In addition, Kruth et al. [30] analysed the effects of the LSR strategy with different process parameters for twice melting the last twenty layers of parts, and obtained a significant roughness improvement and an increase in microhardness and wear behaviour. Other works showed that the tensile stress of the top surface is reduced by 55% when remelting is used for the last layer [32] and only 10% when LSR is used for every layer [33].

The residual stresses occurring during the manufacturing process are a common inconvenience in LPBF parts. Increased residual stresses in AMed alloy exhibit surface defects and dimensional inaccuracies.

Currently, the literature contains limited reports on minimising the edge effects and dimensional accuracy of LPBF parts by varying the laser parameters and scanning strategies. Matache et al. [19] studied the influence of laser power and scanning speed on edge and corner effects in the LPBF process of IN 625 alloy and found that the elevated ridges generated on both specimen sides and corners are strongly influenced by the energy input; additionally, they increase as the laser power is increased and decrease as the scanning speed is increased. Metelkova et al. [20] studied the edge effect of samples after the top surface was remelted from one to ten times with different hatch angles between each remelting layer, keeping the other process parameters constant. They found that as the number of remelting passes increased, the height of the edges decreased and the edge length increased. Yasa et al. [5] found that the edge effect is amplified when a contour scan is used, but that the flatness of the top surface could be further improved by applying raster scanning instead of unidirectional scanning.

There is no possibility of eliminating the edge effect of LPBF parts due to the stress distribution phenomenon [7], but using appropriate process parameters and scanning strategies can improve the flatness of parts [5,19].

A solution for removing or diminishing the edge and corner effects of AMed IN 625 parts may be to reduce the VED for the last layer(s). Another solution that should be considered is the scanning strategy, which may significantly influence the residual stress and deformation of LPBF parts due to variance in local heat distribution [7]. The stripe pattern scanning strategies with an interlayer rotation of 67°, 45° or 90° are often used in an LPBF process. However, various scanning strategies, such as island scanning, cheeseboard, hexagonal, line and rotate scanning at different angles, zig-zag, and in-out and out-in scanning, can be used in an LPBF process [4,7,23,25]. B. Cheng et al. [7] investigated eight of these scanning strategies by a dual simulation-experimental approach and found that the 45° and 67° line-scanning strategy has the minimum stress and deformation values. In contrast, the in-out scanning case has the highest deformation, and there was no significant difference in deformation among line scanning, 45° line scanning, and 45°, 90° and 67° rotate scanning cases.

Kruth et al. [23] investigated the effect of scanning pattern and scanning vector length on the residual stress. They found that the maximum residual stress reduction was achieved when islands rotated 45° from the x-axis. However, the edge and corner effects are not explored thoroughly in the LPBF process, much less the influence of different combinations of scanning strategies, laser parameters, and LSR.

The impact of laser power and scanning speed on the edge and corner effects of IN 625 cubic samples with no contour scan is thoroughly reported in another study [19]. It was found that laser power between 250–300 W and scanning speeds between 0.7–0.8 m·s^−1^ generate a more stable melt with slightly elevated edges and corners. The present study used these experimental data as input data to evaluate the edge effect on the same IN 625 cubic samples using different scanning strategies, laser top surface remelting, and two volumetric energy densities (VED).

## 2. Materials and Methods

For this study, samples of 10 × 10 × 10 mm^3^ were built with a Lasertec 30 SLM (DMG Mori, Bielefeld, Germany) using vacuum gas-atomised IN 625 metal particles (supplied by LPW Technology Ltd., Runcon, UK) as raw material. The IN 625 is one of the most used printable corrosion-resistant materials for high-temperature applications due to its ability to maintain these characteristics even after long exposure to elevated temperatures [34]. The IN 625 powder with the chemical composition presented in Table 1 is predominantly regular, with spherical shaped particles and a size range of 15–45 µm, and the particle size distribution D10 = 20 ± 2 μm, D50 = 30 ± 5 μm, D90 = 45 ± 5 μm experimentally determined by the authors in another study [35].

The Lasertec 30 SLM machine is equipped with a 600 W Yb: YAG fiber laser and has a building volume of 300 × 300 × 300 mm^3^. To avoid metals oxidation, during manufacturing the chamber was flooded with 99.996% pure Argon until a value below 0.2% oxygen was reached. The IN 625 samples were built on a platform preheated to 80 °C using a cross-type support structure with a 3 mm height designed by the software machine (Rdesigner v2019, Realizer GmbH, Borchen, Germany). Sets of two IN 625 cubic samples with contour and no contour scan were built for each variation of the hatch angle rotation, volumetric energy densities (VED) used for the last layer(s), and the number of remelts of the last layer. The first set of two IN 625 cubic samples was built with contour and no contour scans using the same hatching parameters and scanning strategies. The results of optimising the laser parameters to minimise the edge effect are presented elsewhere [19].

The following hatching parameters, which have been selected based on previous work by the authors [19] to reduce the edge effect as much as possible, will hereafter be considered as standard hatching parameters: laser power (250 W), scanning speed (0.75 m/s), layer thickness (40 μm), and hatch distance (0.11 mm). Based on Equation (1), a laser energy input (VED) of 76 J/mm^3^, which will hereafter be referred to as standard VED, was generated by using these process parameters.

(1) $$\mathrm{VED}\;=\;\frac{P}{\nu \;\cdot \;h\;\cdot \;t}\left[\frac{J}{{\mathrm{mm}}^{3}}\right]$$

where P is the laser power (W), ν is the scanning speed (mm/s), h is the hatch distance (mm), and t is the layer thickness (mm).

**Figure 1:**
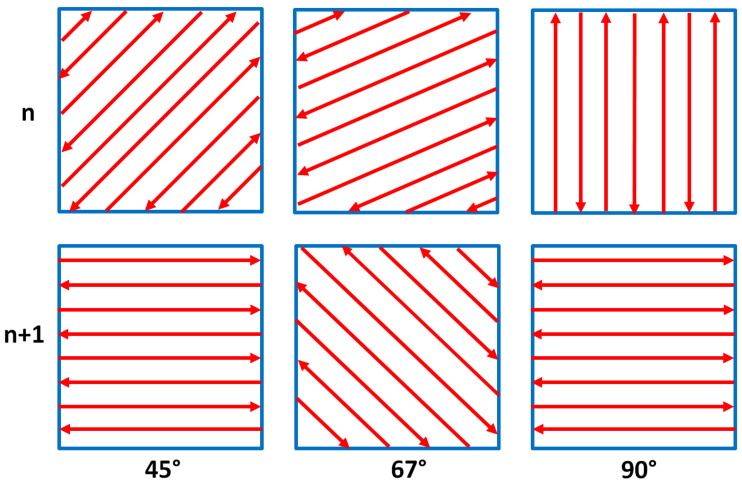
Hatch angles used for manufacturing the IN 625 samples.

For the scanning pattern, three basic hatch angles with rotation of 45°, 67°, and 90° between successive layers schematically shown in Figure 1 were set for both cubic samples with contour and no contour scans. The samples were manufactured according to the standard hatching parameters and contour parameters recommended by the printer manufacturer: laser power (125 W), scanning speed (0.5 m/s), layer thickness (40 μm), fill lines distance 0.1 mm, hatch distance 0.11 mm, and base offset 0.1 mm.

Other sets of samples with no contour scan were built with 45°, 67°, and 90° hatch angle rotations, and were investigated with the last top layer remelted once, twice, and three times. Finally, cubic samples with no contour scan were manufactured using the same hatching parameters and hatch angle rotations but with a lower VED for melting the last layer(s). For this specific lower VED, the laser power was decreased from 250 W to 150 W, while the laser power was increased from 0.75 m/s to 0.9 m/s, generating a lower VED (63 J/mm^3^) than standard VED (76 J/mm^3^) to melt the last one, two, three, five, and ten layers. Each sample was built with no contour scan to eliminate the influence of other factors on the edge and corner effects.

The top-surface topography of the samples (parallel to X–Y plane) was evaluated by 3D laser surface scanning using a coordinate measuring machine NIKON Altera 10.10.8 (LK Metrology Ltd., Derby, UK). The 3D laser surface scanning was equipped with a non-contact NIKON LC15Dx (laser scanning probe with an accuracy of 1.9 µm and an analysis allowance set to 5%). A laser profile with the following process parameters was chosen for scanning the samples: 0.01 mm distance between stripes, 100% laser power, 1% laser exposure, 20% signal threshold, 75° maximum inclination, and 5% edge filter. The edge and corner effects of the samples were quantitatively evaluated by post-processing and analysis of numerically extracted data using dedicated software (FOCUS 2019 R2, Nikon Metrology NV, Leuven, Belgium).

## 3. Results

### 3.1. The Influence of Contour Scan and Hatch Angle Rotation

The elevated ridges that form during the solidification of the alloy reduce the topology and dimensional accuracy of AMed parts. In this study, the evolution of the top surface morphology in terms of elevated ridges and corners was investigated, and different hatch angle rotations and corrective techniques such as laser remelting of the last layer or melting the last layer(s) with a low VED were used to improve the dimensional accuracy of IN 625 samples.

**Figure 2:**
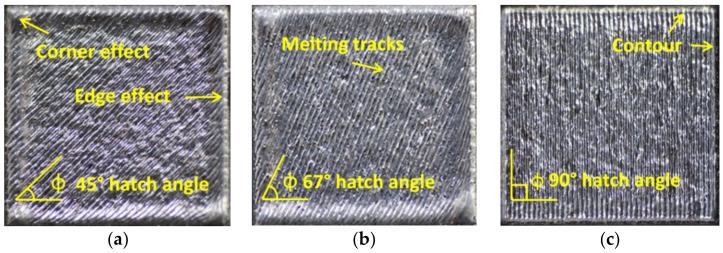
Macroscopic views of the top surface of cubic samples built with contour scan and different hatch angle rotations: (**a**) 45°, (**b**) 67°, and (**c**) 90°.

The top surfaces of samples with contour and no contour built with hatch angle rotations of 45°, 67°, and 90° have been analysed comparatively. Figure 2 presents the macrographs of the top surface of samples built with contour scan and hatch angle rotations of 45°, 67°, and 90° between successive layers.

Figure 2 shows the texture of top surface samples with a contour scan built with 45°, 67°, and 90° hatch angle rotations, consisting of individual melting tracks, contour tracks along the edges, and elevated ridges and corners. During the melting process, the elevated edges and corners are generated due to the surface tension that usually decreases with temperature and tends to drive the melt away from the centre of the melt pool [36]. Due to the layer-by-layer process, the cumulative effects of edges and corners can produce a severe deformation that affects the part’s quality surface [19].

**Figure 3:**
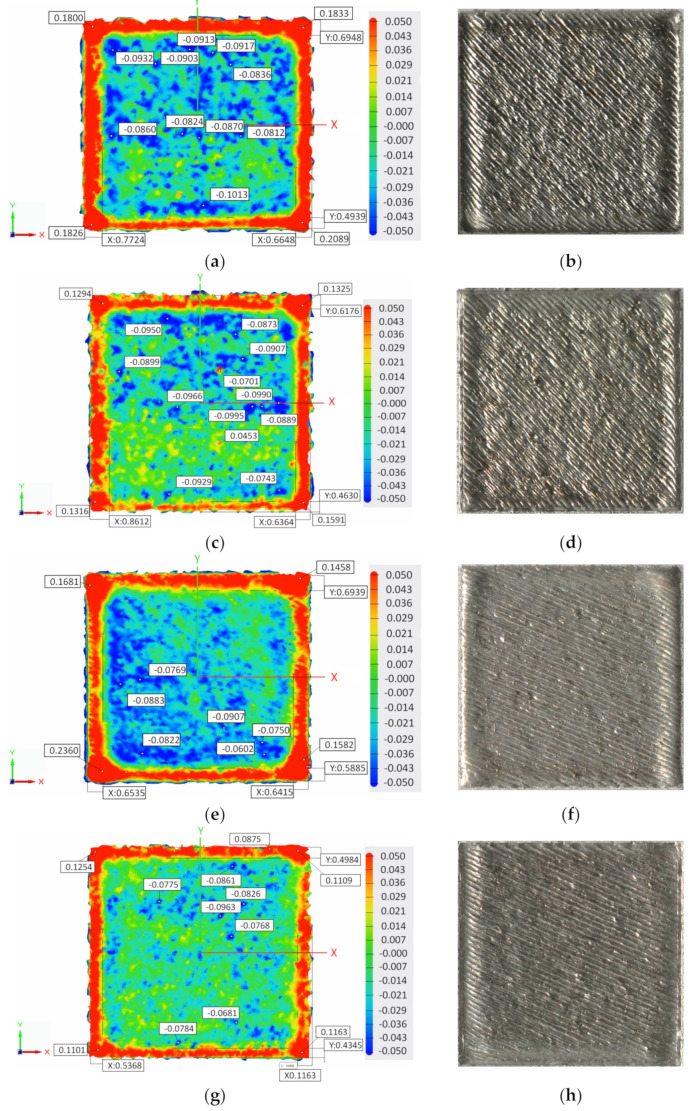

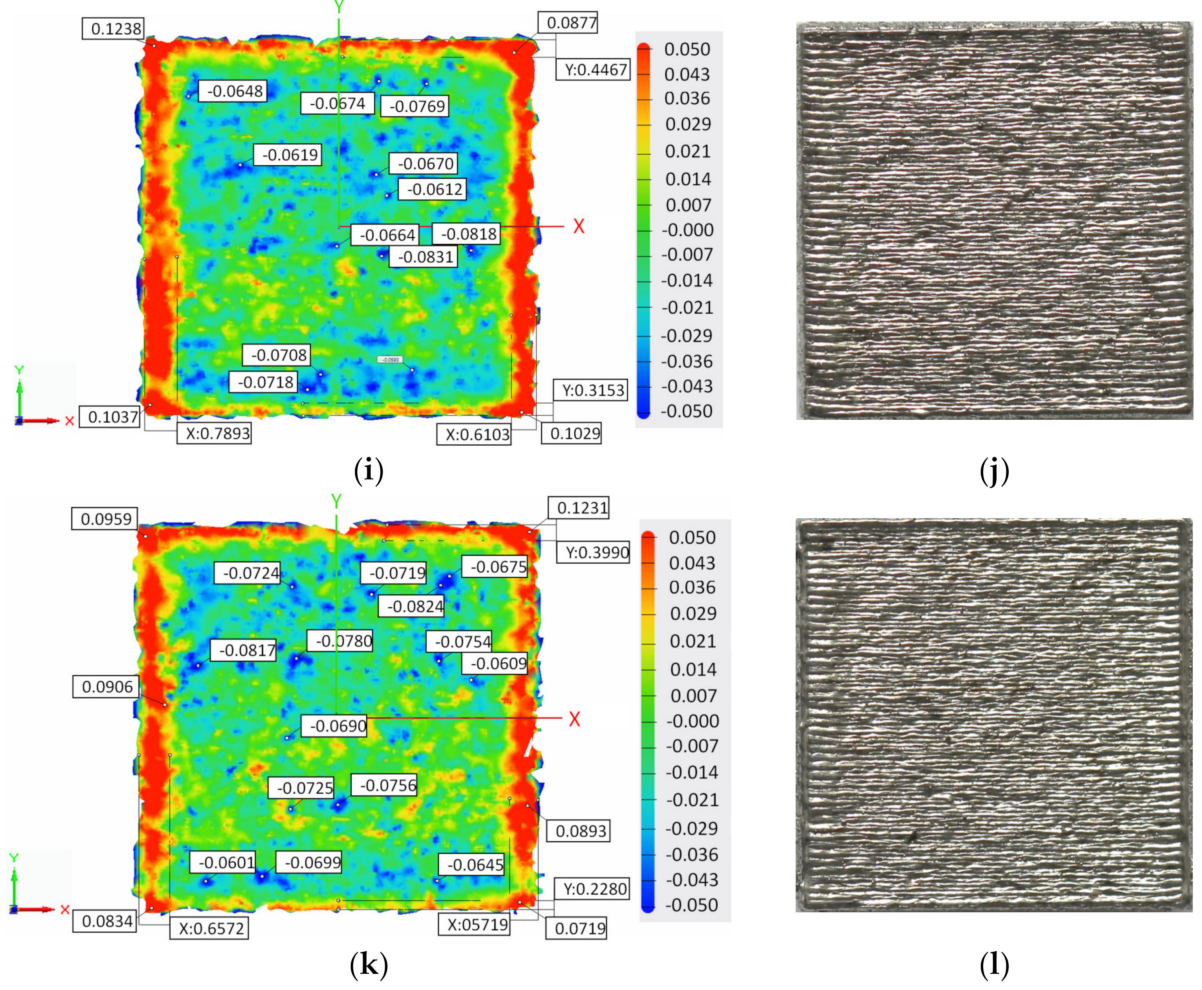
Topography and macroscopic top surface views of samples built with contour and no contour scan using different hatch angle rotations: (**a**,**b**) with no contour and hatch rotation of 45°, (**c**,**d**) with contour and hatch rotation of 45°, (**e**,**f**) with no contour and hatch rotation of 67°, (**g**,**h**) with contour and hatch rotation of 67°, (**i**,**j**) with no contour and hatch rotation of 90°, and (**k**,**l**) with no contour and hatch rotation of 90°.

The corresponding 3D scanning of the top surface of samples with contour and no contour built with 45°, 67°, and 90° hatch angle rotation are presented in Figure 3a–l.

Topographical analysis of top surfaces showed that the elevated ridges on both sample’s sides and corners, generated during solidification, were more prominent when the contour was not used. Even if the cross-section of the sample is square, the corners and edges of the same sample may differ significantly. During the rapid melting and solidification of the alloy, a material build-up occurs due to the compressive stress accumulated around the corners [7] and the thermal warping effect [12]. The scan line being adjacent to the edge of the specimen [3] causes the formation of elevated ridges.

Although the melting tracks are visible in macrographs, the 3D laser surface scanning could not highlight them due to the short hatch distance and small melt pool dimensions. According to a previous study [8], where the morphology and dimensions of the melt pool obtained using the same process parameters as in this study were investigated, the melt pool width and height are 150 ± 17 µm and 86 ± 33 µm, respectively. Furthermore, using a 0.11 mm hatch distance allowed us to achieve a relatively smooth surface, the texture of which could not be detailed by 3D laser surface scanning. However, this study aimed to evaluate the edge and corner effects occurring in AMed IN 625 alloy, and the 3D laser surface scanning has proven to be an effective and reliable technique for measuring the edge effect, as shown in a previous study [19].

To quantitatively measure the edge and corner ridges induced by the contour scan and scanning strategy, the edge and corner heights and edge width were evaluated based on the data extracted from the surface profiles generated by the 3D laser surface scanning. Additionally, the topography top surface views indicated the highest and deepest points and the relative widths of the sample edges (noted with x and y).

**Figure 4:**
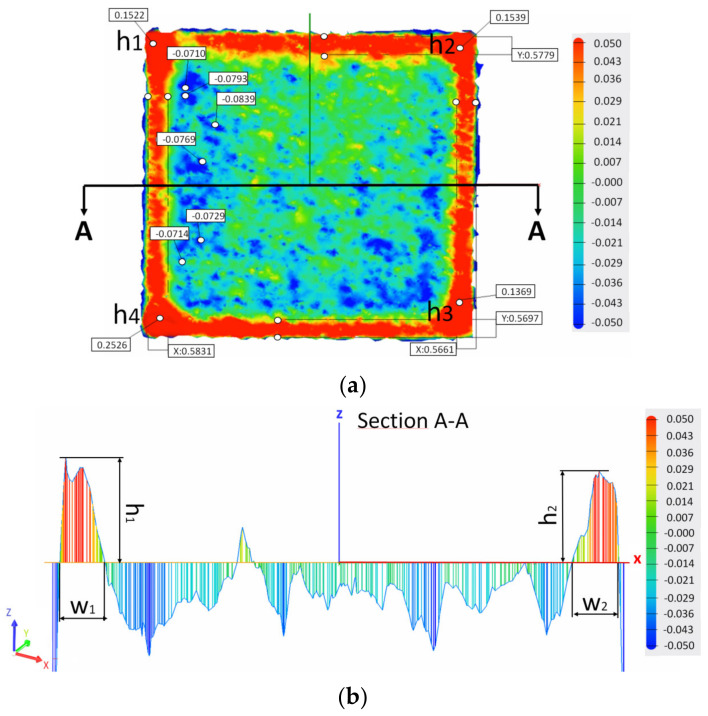
Section used for measuring the edge and corner effects of samples: (**a**) top surface topography of a specimen with the indication of the section and points measured and (**b**) measurement of the edge height and width on the section.

As shown in Figure 4, the corner height (h) was measured as the mean of the four highest points (noted with h1, h2, h3 and h4) from the mean of the flat surface corresponding to the corners of the sample, while the edge height was measured as the maximum height from the mean of the flat surface. In contrast, the edge width (w) was measured as the difference between the side of the specimen and the endpoint where the side ridges end. A relatively high degree of scattering can be obtained when measuring the edge height and width due to the section in which the measurements were performed representing only a tiny area of the specimen, which does not always include the highest points of the edge.

**Figure 5:**
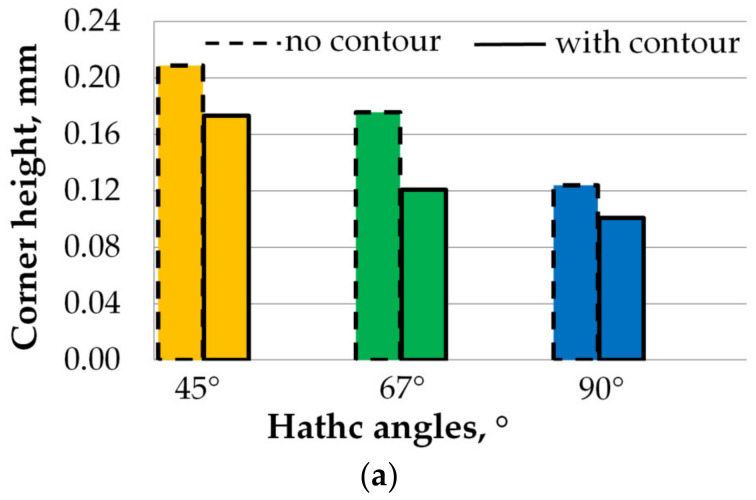

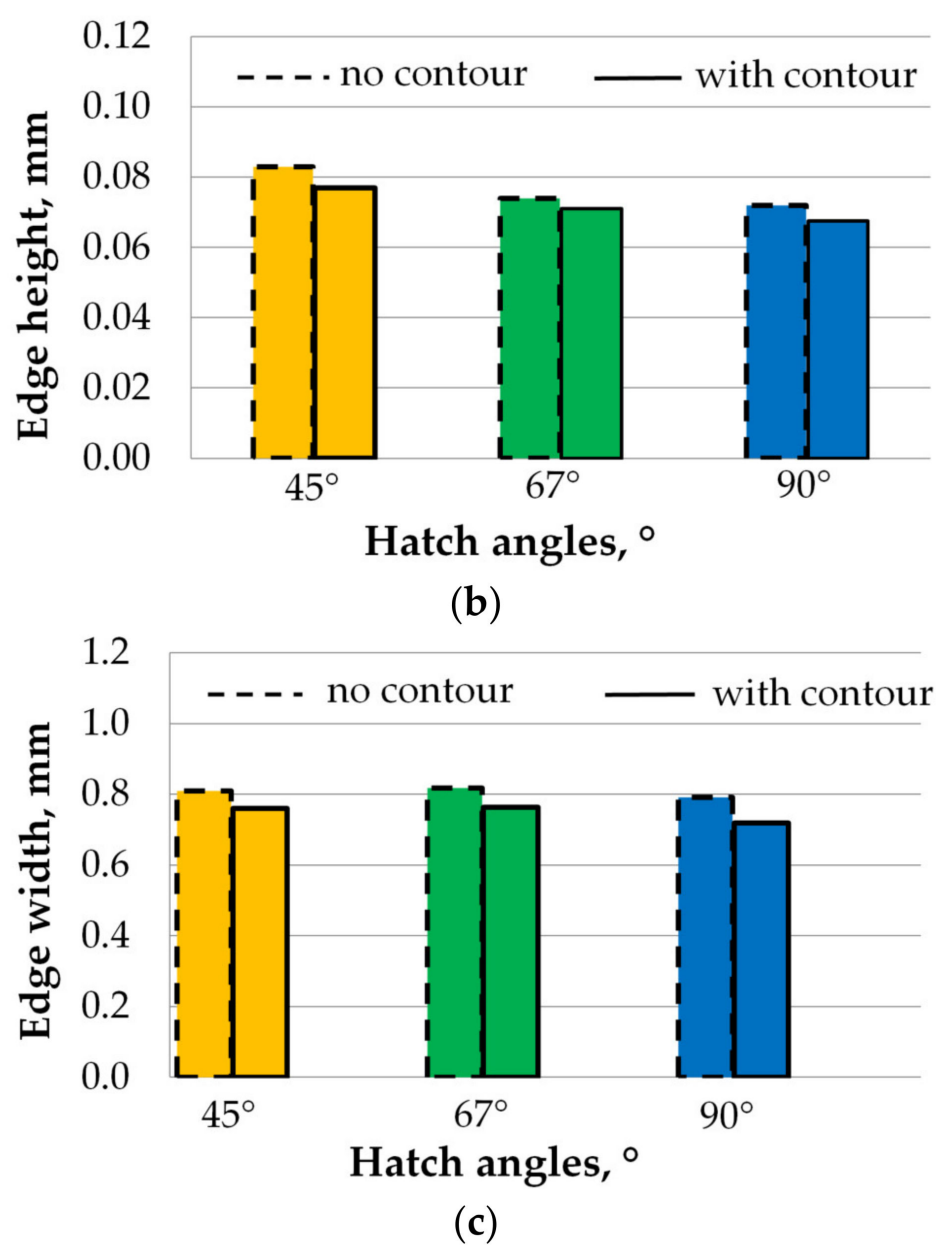
Corner and edge height and width of samples built with contour and without contour as a function of three hatch angle rotations.

Based on the data extracted from the surface profiles of the samples investigated by the 3D laser surface scanning, the quantitative analysis of the edge and corner effects is graphically represented in Figure 5a–c.

The quantitative analysis of the edge effect has revealed that the hatch angle rotation strongly impacts the quality of samples’ top surface by generating elevated ridges on both part’s sides and corners. A global decrease of elevated edges (Figure 5b) and especially corners (Figure 5a) of samples was observed, indicating that using the contour has a corrective action on surface deformation regardless of the hatch angles rotation (45°, 67° or 90°). Manufacturing samples with contour partially solves the deformation problem, but this cannot be used as an ultimate solution, as the dimensional accuracy is highly affected by the corner and edge effects. In all cases, these ridges were more amplified when a 45° hatch angle rotation was used and least amplified when a 90° hatch angle rotation was applied. This behaviour could be related to the accumulated residual stress and higher thermal gradients caused by the 45° and 67° hatch angle rotations that lead to local deformations at the corners and edges of AMed parts. The 90° hatch angle rotation implies a more stable melt pool near the sides of the samples. Due to the epitaxial solidification, a checkerboard pattern is obtained when the scanning between two successive layers is rotated by 90°, while in the cases of scanning by 67° and 45°, the grains are orientated in hexagonal ways.

### 3.2. The Influence of LSR

**Figure 6:**
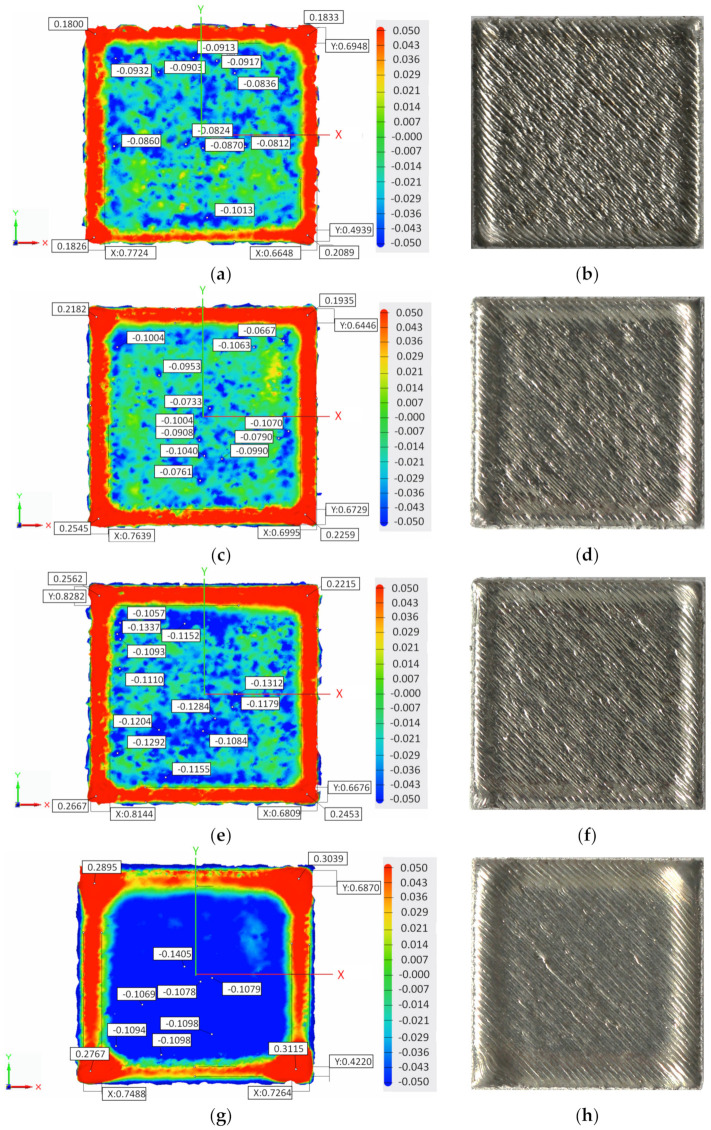
Topography and macroscopic top surface views of the last layer of samples built with a 45° hatch angle rotation: (**a**,**b**) without remelting—reference, (**c**,**d**) with one remelting pass, (**e**,**f**) with two remelting passes, and (**g**,**h**) with three remelting passes.

According to the literature, the LSR strategy was applied to the last layer of cubic samples during the manufacturing process to improve the dimensional accuracy of AMed parts. The LSR strategy was used for remelting the last layer of samples once, twice, and three times using the same standard process parameters and hatch angles rotation (45°, 67° or 90°). The samples were built with no contour scan to avoid the influence of other factors on the edge and corner effects. Examples of the top surface morphologies of samples built with a 45° hatch angle rotation and a remelting of the last layer once, twice, and three times (c) are presented in Figure 6a–h.

**Figure 7:**
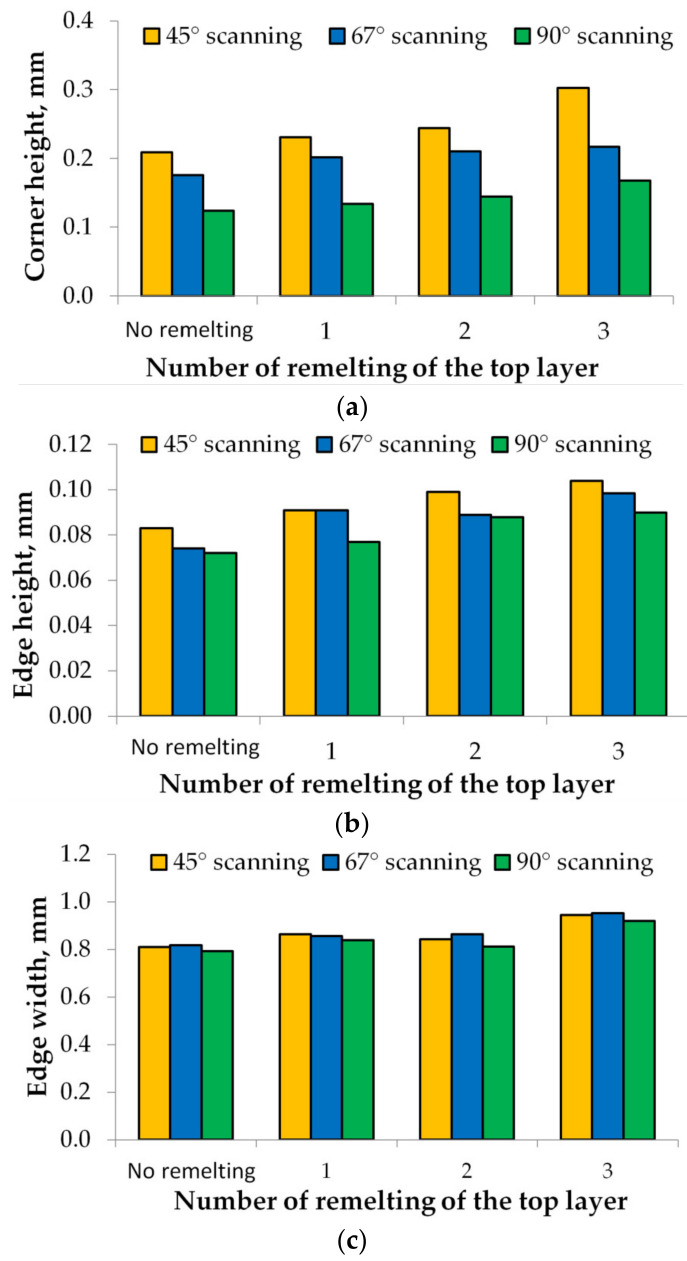
Corner and edge height and width as a function of the remelting passes of the top layer.

The LSR strategy negatively influences samples’ edge and corner heights, regardless of the hatch angle rotation. The surface deformations increase as the remelting passes of the last layer increase, and the corner and edge heights become more prominent, as shown in Figure 7a–c.

The quantitative analysis in Figure 7a–c shows that the elevated ridges increase by decreasing the scan angle rotation and increasing the top layer’s remelting passes. The graph in Figure 7a shows that this behaviour is more pronounced for the corner height, which increases significantly with decreasing hatch angle and increasing number of passes, especially after the third passe. A similar but less pronounced trend was observed for the edge height, as shown in Figure 7b, while the edge width is not significantly affected until after the third remelting passe.

The laser beam pushes the remolten material to the sides, increasing the dimensions of the corners and edges. The main reason for this behaviour may be the too-high VED [11,19]. When the VED used is too high, the molten becomes unstable and generates a recoil pressure that partially pushes the melt to the edge [17,19].

Another reason for the accentuation of the edge effect by the remelting passes of the last layer may be the increase in residual stresses. Generally, lower laser power and higher scan speed are preferred because a lower VED induces a less pronounced edge and corner effect [11,19]; however, it can affect the density, surface roughness and mechanical properties.

### 3.3. The Influence of Using a Lower VED for the Last Layer(s)

**Figure 8:**
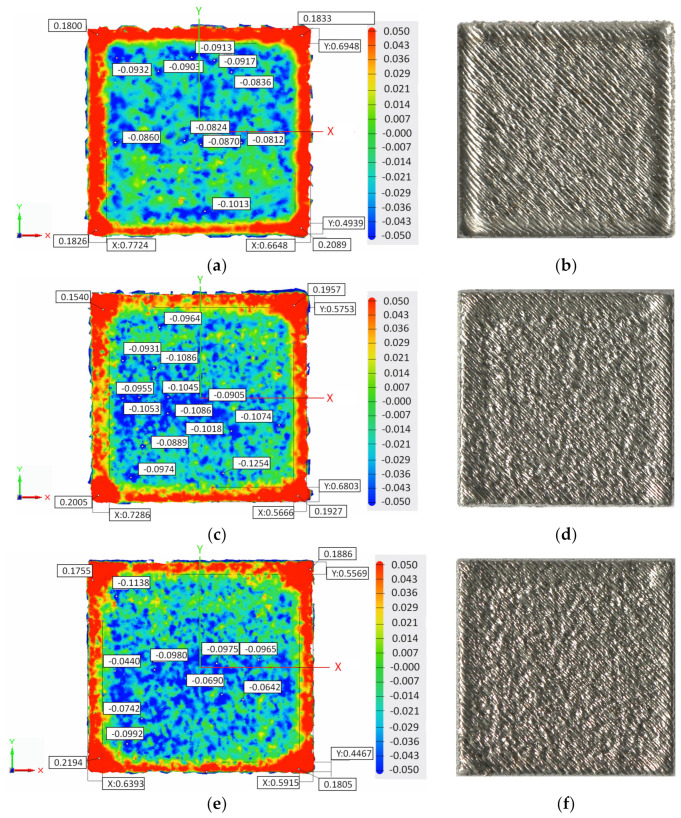

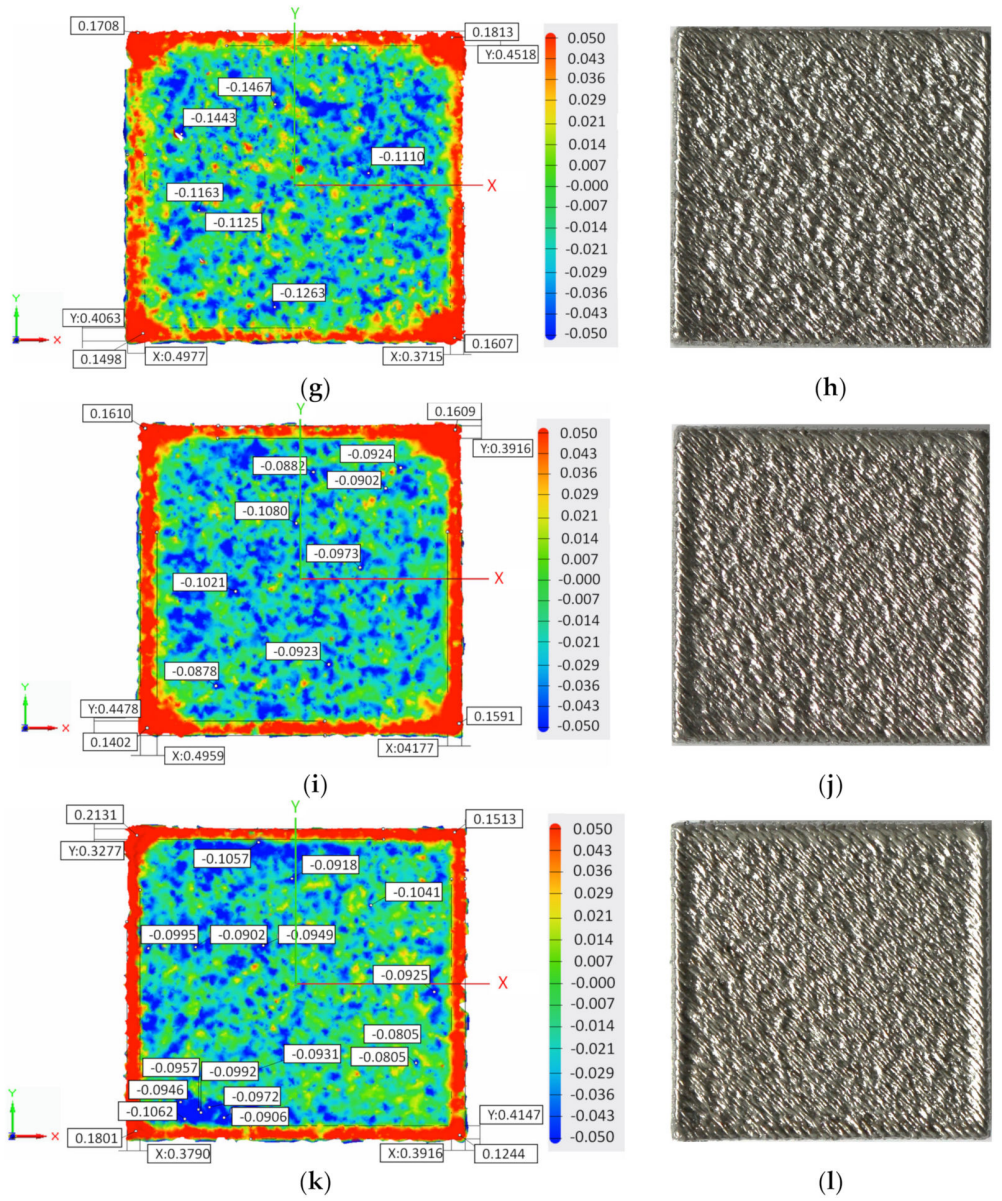
Topography and macroscopic top surface views of the samples built with a 45° hatch angle rotation, using standard VED (76 J/mm^3^) and lower VED (63 J/mm^3^) for melting the last layer(s): (**a**,**b**) standard VED for the last layer, (**c**,**d**) lower VED for the last layer, (**e**,**f**) lower VED for the last two layers, (**g**,**h**) lower VED for the last three layers, (**i**,**j**) lower VED for the last five layers, and (**k**,**l**) lower VED for the last ten layers.

A third case study was to check if using a lower VED for the last layer or several layers is enough to diminish the elevated edges and corners. Therefore, samples with no contour scan were manufactured using the same scanning strategies but with a lower VED (approx. 63 J/mm^3^) than standard VED (approx. 76 J/mm^3^) to melt the last, one, two, three, five, and ten last layers. For this specific lower VED, the laser power was decreased from 250 W to 150 W, while the laser power was increased from 0.75 m/s to 0.9 m/s. This low VED approach should reduce residual stress in the samples. Figure 8a–l presents the top surface of samples built with no contour scan and 45° hatch angle rotation using standard VED and lower VED for the last, one, second, three, five, and ten layers.

**Figure 9:**
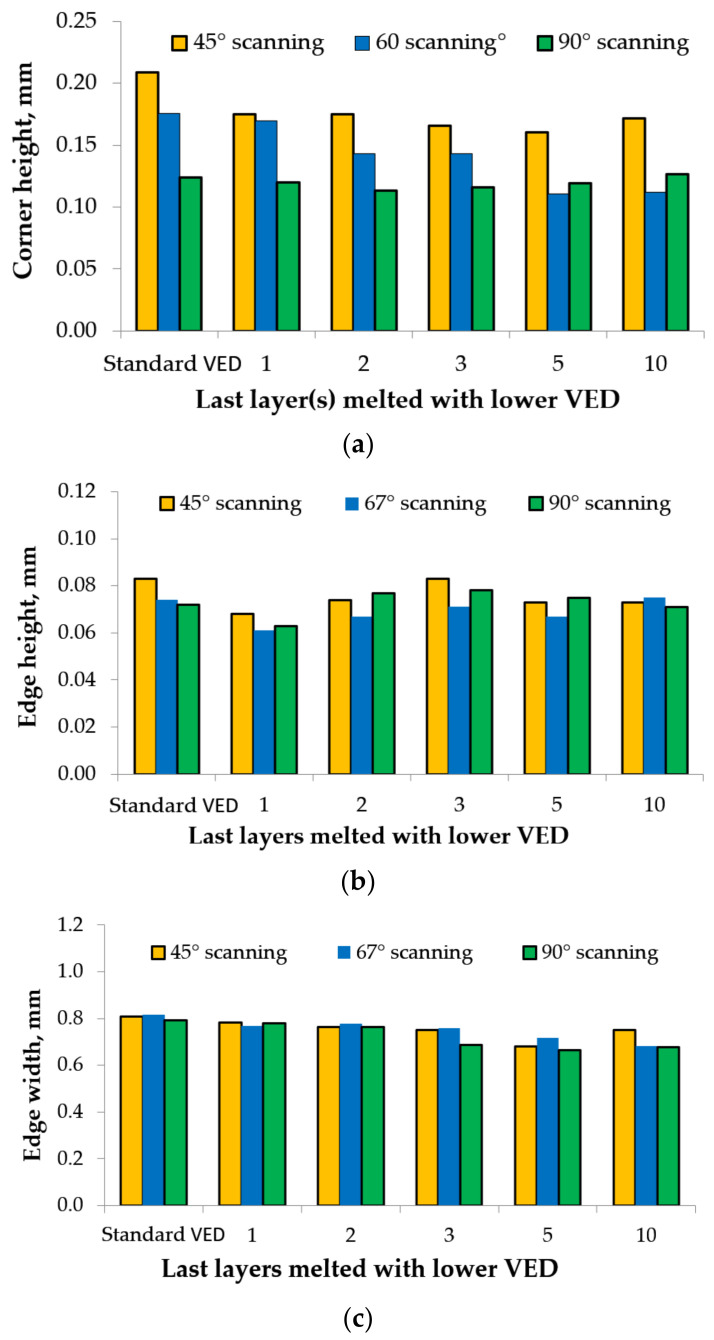
Corner and edge height and width of samples as a function of increasing the number of last layers melted with low VED.

The comparative analysis of the topographic and macroscopic top surface images presented in Figure 8a–l revealed that the edges and corners became thinner using a lower VED. The contour profile became more regular and thinner as the number of last layers melted with low VED increased. Figure 9a–c shows the edge and corner heights and edge width of samples built with standard VED and lower VED for the last one, two, three, five, and ten layers.

The experimental results in Figure 9a–c show that even when using a lower VED rather than a standard VED for melting the last layer, the edge and corner heights were significantly reduced, especially in the cases where samples had high ridges. For example, the edge and corner heights of samples built with hatch angles of 45° were reduced by more than 15% when the last layer was melted with a lower VED (see Figure 9a,b). In the case of edge width, the graph presented in Figure 9c shows that the elevated edge decrease almost linearly as the number of layers melted with low VED increases, both for 45° hatch angle and 67° and 90° hatch angles. The samples built with a lower VED for the last five and ten layers were quite close with respect to elevated ridges, meaning that the edge effect can be reduced only to a certain limit.

Using a lower VED will reduce the size of the melt pool and the volume of material pushed to the edges and corners. The results revealed that the rotation of 45° and 67° hatch angles negatively influences the edge and corner effects even when using a lower VED. However, lower values for the edge and corner heights were obtained using lower laser power and high scanning speed than samples built with a standard VED.

Using a lower VED for melting the last layers generates more acceptable edges and corners heights; however, the surface roughness must be considered because it increases with as the laser power decreases [6]. Moreover, as the number of the last layers melted with a lower VED increases, the mechanical properties, density, porosity, and microstructure may be affected. Therefore, using the lower VED for the last five layers of an LPBF part seems the best compromise to diminish the edge and corner effects when the surface roughness is not a critical requirement of an AMed part.

## 4. Discussion

The present study focuses on reducing the edge and corner effects in additively manufactured IN 625 alloy by using contour scan, different hatch angle rotations, reducing the VED for the last layer(s) of parts, and remelting the last layer once or several times.

The flatness of LPBF samples is generally affected by the raised edges and corners. The first melt sinks into the powder, leading to the volume of solidified material near the pool edge and pushing a larger volume of residual powder to the sides of the parts. Finally, the excess powder will be melted and solidified at the edge and corners of the specimen, increasing their width and height. The melt pool stability and the material flow are governed by the Marangoni convection, which is influenced by the temperature and surface tension gradients [36]. Marangoni convection and recoil pressure influence the increase of melt depth, creating a depression near the melt pool [15,17].

The quantitative assessment of the top layer by 3D laser scanning showed that the samples manufactured with contour scan had lower edges and corners than those with no contour for all hatch angles rotation (45°, 67°, and 90°). The case of corner heights of samples built with contour and hatch angles of 45°, 67°, or 90° were reduced by 17%, 31%, and 19%, respectively, to the same samples but without contour. The contour scan acted correctively on the elevated ridges on both specimen’s edges and corners. The material on specimen sides is partially remelted when the contour technique is used, which facilitates a microstructural refinement effect and a reduction of the surface roughness [12] and, therefore, an improvement of dimensional accuracy.

In general, the contour scan improves the quality surface, especially in the case of inclined surfaces [12]. However, the contour regions may accumulate porosities and voids at the transition zone between contour and bulk scans [12,25], which is more critical than bulk porosity [37]. Instead, Yasa et al. [5] found that the parts built with contour scan had higher ridges than those built with no contour. However, while using the contour scan is mandatory to obtain an adequate dimensional accuracy of AMed parts, it does not completely fix the edge problem of LPBF samples.

The edge and corner effects of specimens built with and without contour were more accentuated when using a hatch angle rotation different than 90°. Generally, the corner height decreases significantly, and the edge height decreases slightly with increasing the hatch angle rotation, while the edge width was not significantly influenced. The corner heights of samples increase by 72% for a 45° hatch angle and 20% for a 67° hatch angle compared to samples built with a 90° hatch angle. Robinson et al. [25] also observed that the deflection of LPBF parts is larger when the scanning strategy is rotated by 45°, and the highest residual stress is generated parallel to the scanned vectors. On the contrary, Cheng et al. [7] found that 45° inclined line scanning generates the smallest deformations of the manufactured parts. However, using a hatch rotation other than alternating 90° does not affect the properties of the AMed part in terms of roughness, strength, density, or residual stresses as long as there is a rotation between successive layers [25].

This behaviour explains that the heat flux direction rotates with the scan between layers, and thus the residual stresses are not equally distributed when using hatch angle rotations of 45°, 67°, or 90°. The elevated edges and corners are produced during the melting process due to the tendency of the melt to push away from the centre of the melt pool as the surface tension decreases with temperature [36]. Furthermore, near the corners and edges of the samples, the temperature profile around the melt pool deviates from the steady-state condition [38].

Another explanation is the crystallographic texture, which is strongly influenced by the crystallographic orientation of the parent grain and scan rotation of every layer [29,38]. It can be assumed that the local deformations at the corners and edges of samples are significantly higher when a hexagonal pattern generated by 45° or 67° scanning is used than when a checkerboard pattern is generated by 90° scanning.

A similar trend was observed when the LSR technique and low VED were used for the last layer(s). The influence of laser remelting once, twice, and three times on the last layer of samples built without contour and with the hatch angle rotation of 45°, 67°, or 90° was investigated in terms of the corner and edge effects.

The 3D scanning results were compared with those obtained on samples built without remelting. The trend of increasing deformation while increasing the hatch angle was also observed when using the LSR technique, but was much more amplified. In the case of samples built with a 90° hatch angle rotation, using the remelting technique increases the heights of the edges and corners by 8% and 7%, respectively, for the first remelting pass; by 17% and 22% for the second remelting pass; and by 35% and 25% for the third remelting pass, compared to the sample without remelting. A similar trend was observed for the other two hatch angle rotations. For the samples built with 67° hatch angle rotation, the heights of the edges and corners increase by 15% and 20% for the first remelting pass, by 19% and 23% for the second remelting pass, and by 23% and 33% for the third remelting pass. In the case of samples built with a 45° hatch angle rotation, the heights of the edges and corners increase by 10% and 10% for the first remelting pass, by 17% and 19% for the second remelting pass, and by 45% and 25% for the third remelting pass.

As the remelting passes increased, the laser beam partially pushed the remelted material to both specimen sides and corners, amplifying the edge and corner effects. A probable cause of this behaviour is the low heat conductivity of powder (in the case of the first melt of the layer) compared to a (re)melted layer. During remelting, a higher surface temperature will be achieved due to the low heat dissipation of the melted layer. After three remelts of the last layer, higher heat conduction and, consequently, high ridges on the top surface will be higher.

These results counter other studies where the LSR is considered a reliable surface finishing method [11,12,26,27] to improve manufactured parts’ surface characteristics and mechanical properties. Although LSR improves density and surface quality, the heightening of the edge effect has also been observed in another work [5]. LSR may be a solution for reducing the surface roughness without significantly impacting manufacturing times, but it has a strong negative impact on the edges and corner effect, as shown in Figure 6. To identify the appropriate process parameters for remelting the last layer, an assessment of the surface roughness and edge effect should be considered. A possible approach to diminish the edge and corner effects may be using the LSR with a lower VED to preheat the metal powder. Aboulkhair et al. [39] use half laser power for the first layer scan and full power for the second layer scan to maximise the relative density of LPBF parts without significantly affecting other properties or characteristics of parts. Based on these results, a new challenge must be considered—optimising the laser process parameters for (re)melting the last layer(s) of LPBF parts.

To reduce the residual stress on the top surface of samples, the layers were built with 250 W laser power and 0.75 m/s scanning speed (76 J/mm^3^), while the last one, two, three, five, and ten layers of samples were melted with 150 W laser power and 0.9 m/s scanning speed (63 J/mm^3^), keeping the other parameters constant.

As was expected, the elevated ridges were significantly reduced compared to the samples built with standard VED, but only to a certain limit. The edge effect reduced as the number of last years increased, but no significant differences with respect to the maximum heights were observed between the samples manufactured with the last five and ten layers with lower VED.

In the samples manufactured with lower VED for melting the last five layers, using 45° and 67° hatch angles considerably reduced the corner heights by 23% and 37%, respectively, while in the case of samples built with a 90° hatch angle, the corner heights were reduced by only 4% compared with standard samples. However, the deformations of samples built with 90° hatch angles and their diminishing effect were generally much smaller than those built with 45° and 67° hatch angles.

The effect of diminishing raised ridges due to reducing laser power and/or increasing scanning speed was also observed in other studies [5,19,40]. Conversely, Yasa [5] found no significant results regarding the edge effect diminishing regardless of varying the laser power and scanning speed. However, using a lower VED to melt the last few layers is beneficial in the reduction of the edge and corner effects with the detriment of top surface roughness, but the number of layers should be kept as low as possible to avoid affecting the mechanical properties, density, porosity, and microstructure of AMed parts.

Therefore, when the surface roughness is not the most critical requirement of parts, using a lower VED for the last five layers of an LPBF part seems the optimal choice to diminish the edge and corner effects without affecting other physical properties.

## 5. Conclusions

The effects of hatch angle rotation, contour scan, volumetric energy densities, and laser top surface remelting on the top surface of LPBF IN 625 parts were investigated in terms of edge and corner height and width based on the data extracted from the surface profiles generated by the 3D laser surface scanning on cubic specimens.

The results showed that the elevated ridges of LPBF samples could not be eliminated, but they can be significantly reduced by using the contour scan. The samples built with hatch angles of 45°, 67°, or 90° and with contour had corner heights reduced by 17%, 31%, and 19%, respectively, compared to the same samples but without contour.

The hatch angle rotation is another scanning strategy that strongly impacted the top surface deformation of AMed IN 625. Compared to samples built with a rotation angle of 90° between successive layers, samples built with hatch angle rotations of 45° and 67° had 72% and 20% higher deformations, respectively.

Using the LSR strategy for the last layer of samples improves the smoothness of the top surface but has no positive impact on the corner and edge effect. The surface deformations increased significantly as the number of remelting passes increased due to the low heat dissipation of the (re)melted layer and high surface tension gradients which generate elevated ridges at the sides of parts. Using the same process parameters for melting and remelting the top layer may have a corrective action on the surface smoothness, but it is not a remedy for reducing the elevated edges of LBPF samples.

An additional possibility that was checked in the present study to improve the flatness of the top surface was to reduce the standard VED (76 J/mm^3^) for melting the last layers. The elevated ridges were reduced when a low VED (63 J/mm^3^) was used for melting the last layer, and generally had a decreasing trend with increasing the hatch angle rotation and the number of the last layers melted. The samples built with low VED for the last five and ten layers were quite close with respect to elevated ridges. Thus, the edge effect can be reduced only to a certain limit. Another approach to diminish the elevated ridges may be using the LSR with lower VED in correlation with an optimal number of remelting passes.

Manufacturing IN 625 samples by LBPF using a lower VED for only the last five layers, a 90° hatch angle between successive layers, and contour technique seems to be the best choice to reduce the edge and corner effects without affecting the manufacturing times and costs.

## Figures and Tables

**Table 1:** Chemical composition of IN 625 metal powder (in wt.%).

Elements (wt.%)	Al	C	Co	Cr	Fe	Mn	Mo	Nb	Si	Ti	Ni
Specification	<0.4	<0.1	<1.0	20–23	3–5	<0.5	8–10	3.15–4.15	<0.5	<0.4	Bal.
Actual composition	0.06	0.02	0.1	20.7	4.1	0.01	8.9	3.77	0.01	0.07	62.26

## Data Availability

Data sharing is not applicable.
